# Prolonged length of stay in the Italian emergency departments: A competing risks frailty model

**DOI:** 10.1371/journal.pone.0361888

**Published:** 2026-11-11

**Authors:** Salvatore Battaglia, Massimo Attanasio, Sabrina Arnone, Agostino Geraci, Giuseppe Murolo, Salvatore Favitta, Marco Enea

**Affiliations:** 1 Department of Economics, Business, and Statistics, University of Palermo, Palermo, Italy; 2 ARNAS Civico G. Di Cristina Benfratelli, Palermo, Italy; 3 Azienda Ospedaliera Universitaria G. Martino, Messina, Italy; 4 AOU Policlinico “G. Rodolico-San Marco”, Catania, Italy; 5 Department of Health Promotion, Mother and Child Care, Internal Medicine and Medical Specialties, University of Palermo, Palermo, Italy; University of Bologna: Universita di Bologna, ITALY

## Abstract

**Background:**

Emergency department (ED) overcrowding has increased worldwide in recent years. This paper provides quantitative information based on data collected immediately before the COVID-19 outbreak, which can serve as a baseline for developing systematic approaches to prolonged length of stay (LoS) in EDs.

**Materials and methods:**

We analysed a random sample of 10,077 ED accesses drawn from the 503,797 eligible accesses recorded in 2019 in the 16 largest hospitals of Sicily, Italy. LoS was modelled using a cause-specific competing-risks Cox frailty model with Discharge and Hospitalization as competing endpoints. Two log-normal frailty components were included to account for unobserved heterogeneity between EDs and between patients. Non-proportionality was addressed by stratifying on pathology and by including time-varying effects for triage. Cumulative incidence functions (CIFs) were computed to estimate the probability of each endpoint and strata over time.

**Results:**

Median LoS was 1.70 hours for Discharge and 3.77 hours for Hospitalization, and the gap widened markedly in the upper tail (90th percentile: 7.10 vs 34.95 hours). Discharge was associated with pathology, age, triage, sex and season; Hospitalization with pathology, age, triage and season. The hazard of Discharge decreased by 2% per additional year of age (HR = 0.98, 95% CI 0.977–0.983) and was 10% higher for accesses in summer (HR = 1.10, 95% CI 1.03–1.16). The hazard of Hospitalization increased by 0.4% per year of age (HR = 1.004, 95% CI 1.001–1.007). The effect of triage was time-dependent: at 10 hours of LoS, red-coded accesses had a 69% lower hazard of Discharge (HR = 0.31) and a 53% higher hazard of Hospitalization (HR = 1.53) than other codes. Circulatory and respiratory diseases and blood diseases showed the highest CIFs for Hospitalization and prolonged LoS for both endpoints, whereas skin and musculoskeletal diseases, injuries and poisoning, and nervous system diseases showed the highest CIFs for Discharge.

**Conclusions:**

Prolonged LoS in Sicilian EDs was driven mainly by pathology, and the exit process differed systematically across disease groups: patients with circulatory and respiratory diseases and with blood diseases waited longest before both Discharge and Hospitalization.The large between-ED heterogeneity in the hazard of Hospitalization, indicates that admission delays reflect organisational as well as structural constraints. Interventions to reduce overcrowding — in particular any expansion of inpatient capacity — should therefore be targeted at the specific wards receiving these patients, rather than applied uniformly across the hospital.

## Introduction

Emergency department (ED) overcrowding has become increasingly prevalent worldwide. It can be defined as a situation in which the function of an ED is compromised, primarily because the number of patients waiting for consultation, diagnostics, treatment, transfer or discharge exceeds the available resources [1]. Despite extensive literature on the topic, ED overcrowding remains a challenging issue, with researchers analyzing its consequences, causes, and associated factors [2–4].

Overcrowding negatively impacts the main actors of the ED: the patient, staff, and system [5]. Patients experienced delayed assessment and care [6,7], more frequent misdiagnosis and medication errors [8–10], longer inpatient length of stay (LoS) [11], higher mortality [12], greater risk of readmission [8] and lower satisfaction [13]; Staff is exposed to stress [8], violence [14] and reduced adherence to best-practice guidelines [15]. These consequences arise from a set of interdependent determinants: a high volume of patients with urgent and complex care needs [16], inadequate hospital capacity [17], shortages and inexperience of ED staff [18,19], and delays in diagnosis and decision-making [20]. Elderly patients often require urgent and complex care. Inadequate hospital capacity (lack of beds in the wards) increases the patient boarding (the number of patients waiting at the ED for hospital admission). Recent evidence confirms that throughput factors such as diagnostic imaging and laboratory testing, together with output factors such as hospital bed occupancy, are the main contributors to prolonged LoS [21]. Late discharges of inpatients and weekend discharges are additional contributing factors [17]. Overcrowding in turn lengthens the time to diagnosis and to exit, so that cause and effect reinforce one another in a feedback loop [22].

However, this literature shows a persistent imbalance. As noted by Morley et al. [5], many studies address the consequences of crowding and its potential solutions, whereas comparatively few address its determinants, and fewer still separate the distinct ways in which patients leave the ED. When more than one type of exit is possible, a competing-risks framework is the natural way to model LoS, because the occurrence of one event precludes the others, and treating the competing events as ordinary censoring biases the estimated event probabilities [23]. Statistically, ED LoS has been modelled with a range of survival approaches, from relative-risk and Cox regression [24–27], while competing-risks models have been applied to ED accesses in a Taiwanese teaching hospital [28] and to burn patients in a national registry [29]. However, often ED data present a multi-centre and repeated-visit structure that has to be taken into account in the model.

In this study, we focus on the Italian setting by estimating a competing risks frailty model for two competing events: Discharge and Hospitalization. The model takes into account both the between-ED variability and the hierarchical structure that arises when the same patient may attend more than once, and patients are nested within EDs. The objective was twofold. First, we aimed to provide quantitative information to policy makers, hospital managers and ED heads on the factors associated with prolonged LoS in the largest Sicilian EDs in 2019, immediately before the pandemic. Second, we proposed a cause-specific hazard model for LoS to predict, through the cumulative incidence functions (CIFs), the two endpoints, for each pathology and time-varying effects to relax the proportional-hazards assumption, incorporating also two frailty components to capture unobserved heterogeneity among patients and among EDs.

## Materials and methods

### Study design and data sources

We used the EMUR (Emergenza Urgenza) database to run the analysis. EMUR is a national administrative database that collects data on patients’ access to EDs in Italy. It is designed to provide financial resources and monitor the quality of medical emergencies in public hospitals, while also ensuring local emergency care. Our analysis is comprised of the Sicilian accesses to the EDs in 2019, which are around 1,700,000. Because ED organisation changed substantially during the COVID-19 outbreak [30,31], data collected immediately beforehand offer an interpretable and stable reference point against which later changes can be read.

For the sake of comparison, we considered just the biggest 16 EDs, including 503,797 accesses. This was done because small hospitals (or small EDs) have different characteristics compared to large ones. Large hospitals encompass all wards related to all medical specialties, so the propensity to choose a large hospital is somehow higher just to avoid being transferred to a large hospital from a small ED. We defined as “eligible” those accesses meeting the following criteria: (i) EDs of general type (we excluded women’s and children’s EDs; as a result, the dataset does not include patients aged less than 15 years); (ii) accesses with complete and valid records (records unidentifiable due to registration errors were excluded); (iii) accesses with outcomes of interest (we excluded patients who died or left the hospital before receiving treatment). Deaths were excluded because they are rare (0.11%), while leaves–-typically “leave before treatment” (6%) and “leave without being seen” (7.6%)–-were excluded because leaving times were not recorded for both types. We considered these accesses to analyze descriptively the LoS.

Each record represents an access, including:

- ED’s variables:
  - Hospital ID hosting the ED;
  - Size: following official documents [32], we classified the Sicilian EDs according to their size defined in terms of the number of accesses in 2019. Our analysis is restricted to EDs with more than 20,000 accesses per year, in order to consider homogeneous EDs. These EDs are located in 7 Sicilian cities – Agrigento, Caltagirone, Catania, Messina, Palermo, Siracusa, and Vittoria (Table 1);
- Patient/Access-level variables:
  - Age: the patient’s age at the moment of arrival at the ED;
  - Sex.
  - Triage level: triage is known to be associated with LoS in the literature [33,34]. Triage presents inconsistencies; specifically, the color distribution varies significantly among the EDs. In other words, there is a large variability among hospitals regarding the conditional probability of Hospitalization and Discharge based on the triage levels. To overcome the classification differences between yellow and green across various hospitals (S1 Table), we opted for a dichotomous classification: *Red level* vs *other level*.
  - Arrival mode: autonomously or by ambulance; this variable is strongly related to *Triage*, since the arrival in ambulance stongly matches with the severe arrivals.
  - Pathology: it has been classified into 11 macro-categories based on clinical criteria using ICD-9-CM codes (S2 Table). For the sake of comparison, we excluded accesses coming from *Complications of Pregnancy, Childbirth, and the Puerperium* (ICD-9 [630–679]), *Congenital Anomalies and Certain conditions originating in the perinatal period* (ICD-9 [740–779]), because they were present just in the EDs belonging to hospitals not having EDs for women and children. We have also excluded *Neoplasms* (ICD-9 [140–239]) because they are few and present different and peculiar characteristics.
  - Calendar arrival time (hour, weekday, season) admission time, and leaving time at the ED. From these calendar times, we have computed: waiting time (*T*1), the time elapsed between arrival and admission; LoS (*T*2): the time elapsed between admission and leaving the ED. LoS is the time of interest used in the survival models, because the relationship of *T*2 or *T*1 + *T*2 with respect to the covariates under study is similar. For simplicity, we categorized Arrival hour in *06 am – 18 pm* and *18 pm – 06 am*; Weekday in *Monday – Thursday* and *Friday – Sunday*; Season in *Summer* and *other seasons*.
  - Frequent users: a binary covariate, equal to 1 if the patient ID appears more than 4 times per year, and to 0 if less or equal than 4 times per year [35].
  - The variables Triage, Arrival Mode, Pathology and Calendar Time may vary according to the access.
- Hospitalization/Discharge decision: these two study outcomes, evaluated over the LoS, are the competing events of interest, as the occurrence of one event precludes the occurrence of the other.

**Table 1:** The biggest Sicilian EDs according to the number of accesses, ordered by initial accesses. Eligible accesses exclude the inappropriate accesses.

EDs	Initial accesses	Eligible accesses	Label
Azienda Ospedaliera Papardo (Messina)	23,473	19,388	1
Ospedale Ingrassia (Palermo)	26,009	25,391	2
Policlinico Gaetano Martino (Messina)	33,612	20,355	3
Presidio Villa Sofia (Palermo)	34,071	30,995	4
Ospedale Vittorio Emanuele (Gela)	34,999	29,493	5
Presidio Ospedaliero Gravina e Santo Pietro (Caltagirone)	36,061	29,427	6
Ospedale Buccheri La Ferla (Palermo)	37,428	33,128	7
Policlinico “Paolo Giaccone” (Palermo)	38,068	37,086	8
Ospedale San Giovanni di Dio (Agrigento)	39,069	33,568	9
Ospedale Guzzardi (Vittoria)	40,628	30,954	10
Presidio Ospedaliero Gaspare Rodolico (Catania)	42,042	38,628	11
Ospedale Cannizzaro (Catania)	49,027	28,081	12
A.R.N.A.S. Ospedali Civico Di Cristina Benfratelli (Palermo)	49,505	47,752	13
Ospedali Riuniti Villa Sofia-Cervello (Palermo)	49,951	20,659	14
Ospedale Umberto I (Siracusa)	51,040	42,286	15
Ospedale Garibaldi (Catania)	53,046	36,606	16
**Total**	**638,749**	**503,797**	

This retrospective study was approved by the Local Ethics Committee Palermo 1 of the A.O.U.P. of Palermo (minutes No. 30, session of 17/12/2025). The Committee approved the scientific use of data from Sicilian emergency department visits for the year 2019. Data were fully anonymized prior to access for research purposes and were accessed on 10/01/2026. Informed consent was not required due to the retrospective design of the study, the use of fully anonymized administrative data, and the fact that the data pertain to a non-recent year.

### Statistical analysis

The descriptive analysis regards the LoS with respect to the two events of interest: Discharge and Hospitalization. We calculated the quartiles plus the $90^{th}$ percentiles, as the Italian regulation suggested that 90% of the accesses have to remain in the ED no more than 24 hours [36].

The structure of the ED data leads to a competing risk setting, where it is reasonable to assume independence between the two endpoints in order to analyze the LoS, denoted by *T*2. The chosen model is the cause-specific hazard model. To account for possible violations of the proportional hazards (PH) assumption, we prepared a person-time format dataset from a random sample of 10,077 records out of the initial 503,797 records (2%). This choice was made to address a computational issue. Specifically, constructing the dataset in the person-time format from the 503,797 records would have generated more than tens of millions records, making the analysis computationally prohibitive (or at least challenging) with our current hardware. To verify that the random sample was representative of the full dataset, we compared the distributions of all covariates and outcomes between the sample and the population. S4 and S5 Tables report the comparison of counts, percentages, and median LoS for Discharge and Hospitalization across covariate categories, along with the standardized difference and the results of the Mann-Whitney test assessing whether sample and population values were significantly different. The comparison showed that the sample characteristics closely mirrored those of the full dataset, confirming that the random sample accurately represents the population.

Our data have a hierarchical structure, where the first-level unit is the patient, as he/she can reappear more than once, while the second-level unit is the ED. Consequently, we employed a multilevel competing risks frailty model to accommodate unobserved differences with patients and EDs. The representation of the model is:


$$\begin{array}{c} \lambda_{iljk}(t|X_{ilj},v_{ik},\gamma_{ilk})=\lambda_{0k}(t)\text{exp}(X_{ilj}^{⊤}\;\beta_{k}+\nu_{ik}+\gamma_{ilk})\\ \text{Where }k=1,2;\;i=1,...,16;\;l=1,..,no.\;of\;patients \end{array}$$ (1)

The quantity $\lambda_{iljk}(t|X_{ilj},v_{ik},\gamma_{ilk})$ is the cause-specific hazard function at time *t* for the $j^{th}$ observation, indicating an access of *t*he $l^{th}$ patient in the $i^{th}$ ED who failed from cause *k*. The log-frailties $\nu_{ik}$ and $\gamma_{ilk}$ are assumed to be normally distributed random effects with zero mean, for the $k^{th}$ competing event. The former term is specific for the $i^{th}$ ED, while the latter is specific for the $l^{th}$ patient of the $i^{th}$ ED. Model estimation was performed by using the *coxph* routine in R with the *frailty* option. The choice of the log-normal frailty distribution enables the random effects to be on the same scale as the covariates [37]. Because only one frailty term is allowed with *coxph*, the procedure that we applied for the estimation of $\beta_{k}$, $\nu_{ik}$, and $\gamma_{ilk}$ was based on the maximization of the log-penalized likelihood according to the algorithm suggested by [38], and firstly proposed by [39].

We assessed the proportionality of the hazard ratios (HRs) by the Schoenfeld residuals, using the *cox.zph* routine in R. To overcome the non-proportionality in relation to the pathology, we stratified by the pathology in both the cause-specific hazard models. For other non-proportional covariates, we included interaction terms between those variables and suitable transformations of time *T*2, by choosing the one providing the lowest BIC value [40]. Finally, estimates of the parameters of the two models and predicted CIFs for each pathology were provided.

## Results

### Descriptive analysis of LoS

Table 2 reports the percentiles of LoS at the ED in hours, calculated on the 503,797 accesses, by access characteristics. Discharge was observed in 78% of the accesses, while Hospitalization in 22%. The median difference in LoS between Discharge and Hospitalization was approximately 2 hours (1.70 hours for Discharge vs 3.77 hours for Hospitalization).

**Table 2:** Percentiles of LoS (hours) at EDs with respect to Discharge or Hospitalization by the covariates present in the access records, in 2019.

	Discharge					Hospitalization				
	p25	p50	p75	p90	# accesses	p25	p50	p75	p90	# accesses
Sex										
Male	0.58	1.68	3.42	7.32	192741	1.72	3.87	11.70	36.63	56699
Female	0.57	1.73	3.41	6.92	201075	1.57	3.68	10.92	33.01	53282
Age										
<30	0.32	1.33	2.62	4.62	90426	0.85	2.43	5.27	15.39	11509
30-50	0.33	1.43	2.87	5.23	122472	1.02	2.70	6.02	18.72	19091
50-80	0.80	2.02	4.07	9.07	151765	1.82	4.15	13.17	39.54	54555
>80	1.50	3.12	7.10	19.97	29153	2.33	5.22	19.53	48.19	24826
Pathology										
Inf_Dis	0.55	1.73	3.37	6.91	9176	1.65	4.00	15.20	43.43	2294
Blood_Dis	2.60	6.49	16.12	28.87	4616	2.56	6.53	23.44	56.00	3404
Circ&Resp_Dis	1.23	2.60	5.40	16.04	29646	1.92	5.13	20.32	50.42	27243
Dig_Dis	0.68	2.02	3.95	8.48	55468	1.92	4.10	10.20	29.50	19557
Nerv_Dis	0.12	1.05	2.43	4.93	53623	0.87	2.60	6.98	22.97	7637
Skin&Musc_Dis	0.63	1.55	2.97	5.12	42375	1.85	3.83	7.35	18.62	5226
Other_Symp	0.85	2.13	4.07	7.88	109112	1.50	3.27	7.68	24.42	27577
Inj&Pois	0.47	1.33	2.50	4.62	87715	1.52	3.20	10.43	29.83	16596
Season										
Winter	0.65	1.80	3.58	7.67	89364	1.77	4.10	13.53	41.86	27684
Spring	0.58	1.70	3.45	7.20	98273	1.63	3.75	11.60	38.41	27676
Summer	0.47	1.62	3.27	6.62	106387	1.53	3.50	9.73	29.03	26965
Fall	0.62	1.72	3.42	7.05	99792	1.65	3.77	10.86	30.47	27656
Weekday										
Mon – Thu	0.60	1.73	3.47	7.13	234068	1.63	3.85	11.71	31.93	65575
Fri – Sun	0.55	1.67	3.35	7.03	159748	1.63	3.65	10.80	39.03	44406
Arrival hour										
06 am – 18 pm	0.63	1.85	3.67	6.73	129903	1.88	4.23	8.83	31.37	33950
18 pm – 06 am	0.55	1.65	3.28	7.41	263913	1.55	3.55	13.13	37.32	76031
Arrival Mode										
Ambulance	1.53	3.07	6.77	18.31	58505	2.15	4.87	17.43	45.77	43208
Autonomous	0.43	1.53	3.05	5.08	335311	1.33	3.25	8.27	26.63	66773
Triage										
White	0.10	0.70	1.80	3.00	34202	0.35	1.98	4.98	23.32	1006
Green	0.28	1.30	2.58	4.57	237981	0.95	2.75	6.42	20.32	26747
Yellow	1.72	3.10	6.15	14.45	115961	2.00	4.28	13.62	39.27	69123
Red	2.75	6.15	18.14	40.47	5672	1.38	3.88	18.00	47.53	13105
Frequent user										
No	0.58	1.70	3.40	7.00	361825	1.65	3.77	11.38	35.15	101734
Yes	0.42	1.68	3.40	8.27	31991	1.30	3.77	10.66	32.97	8247
Total	0.57	1.70	3.42	7.10	393816	1.63	3.77	11.33	34.95	109981

LoS = Length of Stay; ED = Emergency Department; p25, p50, p75, p90 = 25th, 50th, 75th, and 90th percentiles.

The LoS tended to increase with age. Moreover, the difference in LoS between Discharge and Hospitalization also increased with age. For patients younger than 30, the median difference in LoS was around 1 hour, while for patients older than 80, it was around 2 hours. This difference further increased as we move up the percentiles.

The LoS is also strongly influenced by the pathology, with blood diseases (Blood_Dis) and circulatory and respiratory diseases (Circ&Resp_Dis) exhibiting the highest LoS. The largest discrepancy in LoS was observed for infectious diseases, where at the $90^{th}$ percentile, the LoS for Hospitalization was 37 hours longer than for Discharge.

Patients arriving by ambulance generally had a higher median LoS compared to those who came autonomously. Additionally, the difference in LoS between Discharge and Hospitalization was larger for patients arriving by ambulance (1.80 hours in the median, and around 27 hours at the $90^{th}$ percentile).

Finally, patients with triage red level had the highest median LoS (6.15 hours for Discharge; 3.88 hours for Hospitalization).

Regardless of the outcome, no large differences in LoS were observed between frequent users and non-frequent users, between females and males, among hours, and weekdays.

### The competing risks frailty model

By indicating with *D* = Discharge and *H* = Hospitalization the two competing events, the two initial cause-specific PH frailty models, including the significant variables, were:


$$\begin{array}{cc} \lambda_{ijlD}(T2|X,\nu_{iD},\gamma_{ilD})= & \lambda_{0D}(T2)*\text{exp}(Age_{ijl}\beta_{1D}+Sex_{ijl}\beta_{2D}+Triage_{ijl}\beta_{3D}+\\ & Season_{ijl}\beta_{4\;D}+{\text{Pathology}}_{ijl}^{T}\;\beta_{5D}+\nu_{iD}+\gamma_{ilD}) \end{array}$$ (2)

$$\begin{array}{cc} \lambda_{ijlH}(T2|X,v_{iH},\gamma_{ilH})= & \lambda_{0H}(T2)*\text{exp}(Age_{ijl}\beta_{1H}+Season_{ijl}\beta_{2H}+\\ & Triage_{ijl}\beta_{3H}+{\text{Pathology}}_{ijl}^{T}\;\beta_{4H}+\nu_{iH}+\gamma_{ilH}), \end{array}$$ (3)

where **Pathology** is a double vector, and $\beta_{5D}$ and $\beta_{4H}$ are the corresponding vectors of coefficients.

*Checking the PH assumption*. In the model for Discharge, the covariates that violated the proportional hazards assumption are pathology, sex, season, and triage; while in the model for Hospitalization are age, triage, and pathology (S3 Table).

To address the non-proportionality of pathology, we built two stratified hazard models using pathology as the stratification variable, assuming it identifies distinct subpopulations. Additionally, we specified the time-varying effect of *Triage* by including additional interaction terms of *Triage* with a function of *T*2 to overcome non-proportionality in both models: Triage*ln(T2) in the model for Discharge and $Triage*T2+Triage*T2^{2}$ in the model for Hospitalization.

The final models are:


$$\begin{array}{c} \lambda_{ijlD}(T2|X,v_{iD},\gamma_{ilD})=\lambda_{0zD}(T2)\text{ exp}(Age_{ilj}\beta_{1D}+Sex_{ilj}\beta_{2D}+\\ Triage_{ilj}\beta_{3D}+Triage_{ilj}*\ln(T2)\beta_{4D}+Season_{ilj}\beta_{5D}+v_{iD}+\gamma_{ilD}) \end{array}$$ (4)

$$\begin{array}{c} \lambda_{ijlH}(T2|X,v_{iH},\gamma_{ilH})=\lambda_{0zH}(T2)\text{ exp}(Age_{ilj}\beta_{1H}+Season_{ilj}\beta_{2H}+\\ Triage_{ilj}\beta_{3H}+Triage_{ilj}*T2\beta_{4H}+Triage_{ilj}*T2^{2}\beta_{5H}+v_{iH}+\gamma_{ilH}), \end{array}$$ (5)

where *z* = 1,...,8 is the stratum. The quantities of interest provided by the models, that is the estimated fixed and time-varying effects, the frailty components, and the estimated CIFs are described as follows.

*Fixed and time-varying effects.* Table 3 reports the model estimates for both Discharge and Hospitalization. The baseline profile refers to a hypothetical 52-year old (the mean age) with non-red triage level, not accessing in summer. The sex, even if slightly significant, was included in the model for Discharge but not for Hospitalization. Concerning time-varying effects, the signs of the main effect of *Red level* and its interaction with LoS are discordant in both models (Table 3). For a correct interpretation of the model estimates, the overall HRs are computed when LoS is equal to 10 hours.

**Table 3:** Parameter estimates with 95% confidence interval from the Cause-specific competing-risk frailty model stratified by pathology, and frailty variances (ED-level: ${\hat{\sigma }}_{\nu }^{2}$; patient-level: ${\hat{\sigma }}_{\gamma }^{2}$). Baseline profile: median age (54); sex (male); season (other); triage code (other). Hazard Ratios (HR) are reported only for main effects.

Discharge						Hospitalization					
Variable	Coef.	SE	Lower	HR	Upper	Variable	Coef.	SE	Lower	HR	Upper
Age	−0.02	0.007	0.977	0.981	0.983	Age	0.004	0.001	1.001	1.004	1.007
Sex F	0.08	0.028	0.98	1.05	1.10	Season Summer	−0.10	0.05	0.82	0.91	1.01
Triage Red	−2.06	0.18	0.09	0.13	0.16	Triage Red	0.83	0.12	1.81	2.30	2.93
Triage Red × ln(*T*2)	0.38	0.09	.	.	.	Triage Red × *T*2	−0.05	0.02	.	.	.
Season Summer	0.09	0.032	1.03	1.10	1.16	Triage Red × *T*2^2^	0.001	<0.001	.	.	.
${\hat{\sigma }}_{\nu }^{2}$	0.052					${\hat{\sigma }}_{\nu }^{2}$	0.294				
${\hat{\sigma }}_{\gamma }^{2}$	0.494					${\hat{\sigma }}_{\gamma }^{2}$	0.245				

HR = Hazard Ratio; CI = Confidence Interval; ED = Emergency Department; LoS = Length of Stay; SE = Standard Error. The Hazard Ratios are computed assuming the exponential of the interaction terms equal to 1.

In the model for Discharge, the hazard decreases by 2% per 1-year increase in *age* (HR = 0.98). After 10 hours of LoS, the hazard of being discharged for *Red level* is less than 69% compared to *other levels* (HR(LoS = 10) = exp(−2.06 + 0.38 ln (10)) = 0.31). The hazard of being discharged for those accessing in *Summer* is higher than 10% compared to *other seasons*.

In the model for Hospitalization, the hazard increases by 0.4% as *age* increases by one year (HR = 1.004). After 10 hours of LoS, the hazard of being hospitalized is 53% higher for *Red levels* compared to *other levels* (HR(LoS = 10) = exp(0.83–0.05*10 + 0.001*10^2^) = 1.53).

*Frailties*. The ED-level ($\chi_{D}^{2}$ = 392, p < 0.001; $\chi_{H}^{2}$ = 421, p < 0.001) and the patient-level ($\chi_{D}^{2}$ = 414, p < 0.001; $\chi_{H}^{2}$ = 473, p = 0.03) frailty components are significant in both models, and 11 out of 16 hospital effects are significant, with confidence intervals larger for hospitalization. This is sometimes due to the sample size, but, in general, is due to longer LoS for Hospitalization (S1 and S2 Figs). The lowest random effects for Hospitalization are registered in the four Palermo’s EDs. The random effects signs are concordant between the two outcomes in 9 EDs. Among them, 4 EDs show positive effects, suggesting quicker decision processes compared to the median hospital, while, the other 5 EDs display negative effects, indicating slower decision processes.

*Cumulative incidence functions.* Starting from the estimated baseline hazards ${\hat{\lambda }}_{0zD}(T2)$ and ${\hat{\lambda }}_{0zH}(T2)$, we computed the model predicted CIFs with random effects equal to 0 for both events.

Fig 1 shows that Discharge CIFs for most pathologies reach a plateau around 10 hours, indicating that the majority of patients who are going to be discharged leave the ED within the first 10 hours. An exception is represented by Blood_Dis and Circ&Resp_Dis, whose discharge CIFs continue to rise more slowly beyond 10 hours, reflecting prolonged LoS before discharge.

**Fig 1:**
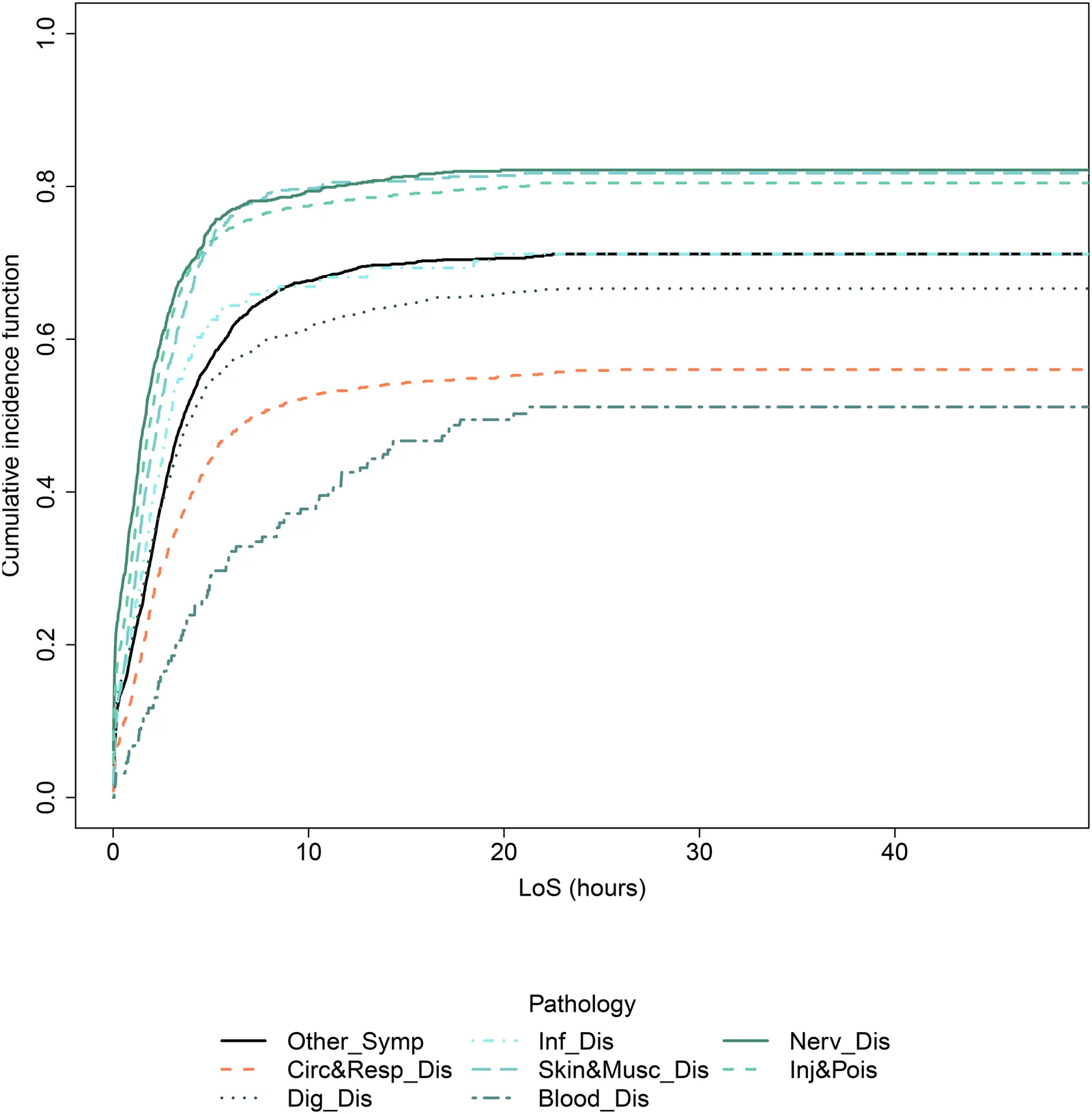
Discharge CIFs by pathology, predicted for the baseline profile, with respect to LoS.

Fig 2 presents the Hospitalization CIFs, which generally reach a plateau only after approximately 20 hours, suggesting that hospitalization decisions tend to require more time than discharge decisions.

**Fig 2:**
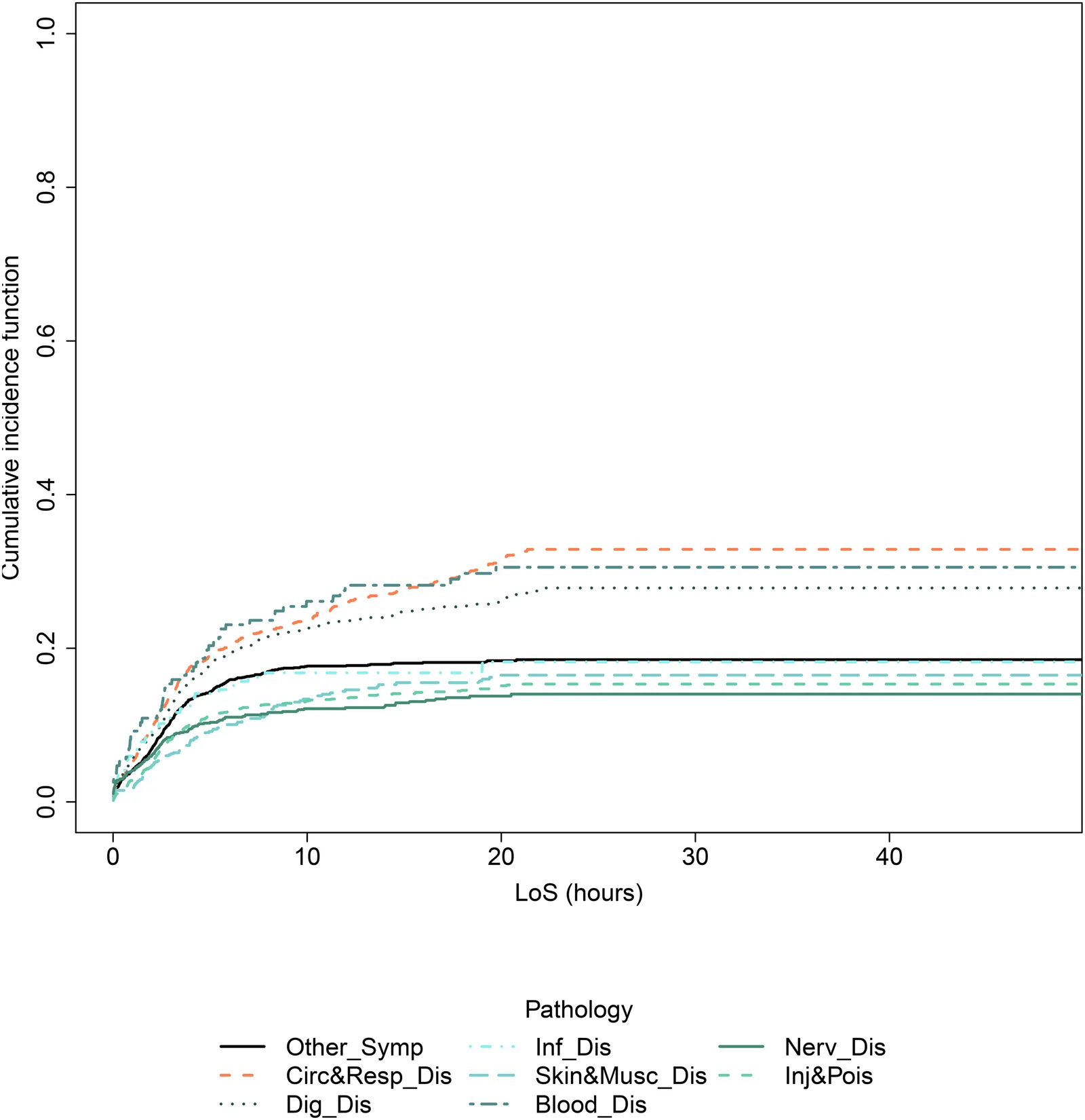
Hospitalization CIFs by pathology, predicted for the baseline profile, with respect to LoS.

Circ&Resp_Dis and Blood_Dis emerge as the two pathologies most strongly associated with prolonged ED stays. At 10 hours, the Discharge CIF for Circ&Resp_Dis is 0.50, for Blood_Dis is 0.45, and for skin and musculoskeletal diseases (Skin&Musc_Dis) is 0.85. At 30 hours, these values reach 0.52, 0.55, and 0.85, respectively. For Hospitalization, at 10 hours the CIF for Circ&Resp_Dis is 0.32, for Blood_Dis is 0.27, and for Skin&Musc_Dis is 0.10. These patterns indicate that blood and circulatory/respiratory diseases are associated with the longest LoS for both Discharge and Hospitalization, whereas skin and musculoskeletal diseases show the shortest LoS. Other pathologies exhibit intermediate CIF values between these extremes. The CIFs along with confidence intervals for Circ&Resp_Dis and Skin&Musc_Dis are reported in the Supplementary material (S3 and S4 Figs).

## Discussion

This study represents the first application of a statistical approach to LoS in Sicilian ED data.

The main findings of our study can be summarized as follows. Pathology was strongly associated with LoS, with circulatory and respiratory diseases and blood diseases showing longer LoS for both Discharge and Hospitalization. Triage was also related to LoS, although its effect was strongly time-dependent: the effect of a red code was more pronounced during the first hours of the ED stay and gradually decreased thereafter. Both patient-level and ED-level frailty components were significant, indicating large unobserved heterogeneity among patients and across EDs, with greater ED-level variability for Hospitalization than for Discharge. Finally, age was associated with a higher risk of Hospitalization and a lower risk of Discharge, while the effect of Sex was weak and the seasonal pattern showed opposite direction of effect for the two events. Overall, these findings highlight the importance of distinguishing between the two competing exit pathways and of accounting for both patient- and ED-level heterogeneity when analysing ED LoS.

The results are in line to the evidence that ED overcrowding is a complex phenomenon and there is no single set of determinants that applies to all healthcare systems. Because healthcare systems are organised at the national and regional levels, the relative contribution of different factors can vary considerably between countries, and solutions need to take local conditions into account. Jayaprakash et al. [41] noted that despite differences between the US and European healthcare systems, ageing populations, limited hospital resources, staff shortages and delays in ancillary services are common sources of pressure. They also highlighted the role of public funding in determining factors such as hospital bed availability and staffing levels.

In Italy, the Ministry of Health has issued policy directions that hospitals are expected to follow, including the use of dedicated indices to monitor overcrowding [42–44], with measurements recorded at least every 24 hours [36]. At the same time, part of the organisation of healthcare is delegated to the regions, which can lead to differences in the way ED accesses are recorded. Previous Italian studies have examined ED utilisation among chronic and frequent users using latent-class models [45], while others have linked seasonal variations in admissions, particularly during winter, to population characteristics and patient acuity [46].

Our analysis refers to the Sicilian EDs immediately before the COVID-19 pandemic, and recent Italian evidence suggests that pressure on EDs has increased since then. Antonazzo et al. [47] analysed 3,563,565 accesses to the 16 EDs of the Abruzzo region between 2017 and 2024 and found that waiting time and process time were broadly stable up to 2020, but increased progressively from 2021 onwards. Mean process time increased from approximately 200 minutes in the pre-pandemic period to 346 minutes in 2024, while mean waiting time reached 78 minutes. A similar increase in total ED LoS was reported by Stirparo et al. [48] in the provinces of Milan and Monza, where the average stay in 2022 was substantially longer than in 2019. In this context, our 2019 Sicilian data provide a picture of the exit process before the more recent increase in pressure on EDs. Antonazzo et al. [47] also found longer waiting and process times in the four largest hub hospitals of their region compared with smaller facilities. Our study cannot fully address this comparison, partly because we restricted the analysis to EDs recording more than 20,000 accesses per year. The comparison among the 16 EDs was instead based on an ED-level frailty component, which revealed substantial heterogeneity even among large EDs, particularly for Hospitalization.

Another novel methodological contribution concerns the way LoS has been analysed with Italian ED data: most Italian studies have considered waiting or process time as a single outcome, whereas our competing-risks approach separates the two possible exit pathways. These pathways can show opposite associations with the same covariate: as expected, factors associated with faster Discharge may be associated with slower Hospitalization. Analysing total LoS alone could therefore mask these differences. Outside Italy, there are many studies addressing this issue. For instance, Pines et al. [24] used relative-risk regression, while Sprivulis et al. [25] applied a Cox model. Both approaches treated LoS as leading to a single type of exit and therefore did not distinguish between discharge and admission. Harris et al. [26] subsequently used a parametric survival model for time to admission, allowing the role of access-block factors to be examined, but still without accounting for competing events. Kheirbek et al. and Chaou et al. [27,28] explicitly introduced competing risks. In particular, the latter applied an accelerated failure-time model and identified age, triage, transfer and arrival time as relevant factors. However, both studies were conducted in a single centre and did not account for unobserved heterogeneity. Taylor et al. [29] also used a cause-specific competing-risks approach, considering Death and Discharge as competing events in burn patients, but their analysis was based on a single registry. Rueten-Budde et al. [49] proposed a competing-risks frailty model to account for hospital heterogeneity, using an EM algorithm to estimate a single frailty term in a Bayesian framework.

Our approach extends these previous applications by combining competing risks with two frailty components: the first, at patient level, as a patient can have several accesses, and the second, at ED level. We found that age and triage were also important in our multi-centre setting, in agreement with Chaou et al. [28], and we were additionally able to quantify variation among EDs and among patients. Moreover, the estimation of the frailty components employs penalised likelihood, providing a relatively direct way of accounting for the two different sources of unobserved variation.

The main findings of the paper are now summarized, taking into account the substantive meaning of the covariates included in the models, in relation to their analytical formulation specified in the competing-risks equations. They are:

*Pathology*. Our data show that Pathology is strongly associated with LoS, although the literature has generally focused on other covariates, most of which can be related to the well-known “Input, Throughput, and Exit” framework. To overcome the non-proportionality, we inserted Pathology as a stratification variable with eight strata in both competing-risks models. Circulatory and respiratory diseases and blood diseases were associated with longer LoS before both competing endpoints, suggesting that the wards receiving these patients may represent important sites of incoming block. It is important to clarify that pathology per se is not a risk factor associated with longer LoS. Rather, the LoS differences may depend, on the one hand, on hospital organization in terms of diagnostic procedures and testing and, on the other hand, on the availability and boarding processes across the different hospital wards. Unfortunately, we do not have information on the variables associated with these processes.

*Triage*. Triage was another important risk factor for LoS, and its effect changed over time in both models, being strongly time-dependent for both endpoints. In particular, the priority assigned by a red code affected the hazard of both exits mainly during the first hours of the stay, with a progressively weaker effect thereafter. For Discharge, the main effect of a red code was strongly negative, while its interaction with ln(T2) was positive. Thus, the hazard of discharge for red-coded accesses was much lower than for the other codes in the first hours, but the difference decreased subsequently, resulting in a hazard ratio of 0.31 at 10 hours of LoS. The pattern for Hospitalization was opposite. The main effect of a red code was positive, but this effect was progressively reduced by the negative linear interaction with T2, with a small positive quadratic term partly attenuating this decrease. The resulting hazard ratio at 10 hours was 1.53. This finding suggests that indicators based only on triage may be useful for monitoring the early stages of ED care, but become less informative as the stay progresses. We also observed substantial variability among EDs in the assignment of triage colours, particularly for green and yellow codes (S1 Table). For this reason, we used a dichotomous red-versus-other classification. This variability is consistent with recent Italian evidence. Nurchis et al. [50], analysing 56,284 accesses using a causal-mediation approach, found that triage code was a strong predictor of one-year mortality, largely independently of waiting time.

*Frailty*. The two frailty components provide useful information about the sources of variability in ED LoS. The patient-level term was included to account for frequent users, while the ED-level term reflects the multi-centre structure of the data. Both components were significant in the two models. The patient frailty variance was higher for the discharge group. This is due to the fact that the LoS in the discharge group varies more (among frequent users) than in the hospitalization group.

As expected, the ED-level variance was higher for Hospitalization than for Discharge. One possible explanation is that hospitalization involves a more complex process and depends on several organisational factors, including inpatient bed availability, specialist consultations, additional diagnostic tests, ward workload and coordination between the ED and the receiving ward. The discharge process is generally less dependent on these steps and may therefore be more similar across EDs. The random-effect estimates were concordant between the two outcomes in nine EDs. The four Palermo EDs also showed the lowest random effects for Hospitalization, suggesting comparatively slower admission decisions in these facilities (S1 and S2 Figs).

*Age, Sex, and Season*. The results related to these three risk factors are in line with what was expected: the HR for age is greater than 1 for Hospitalization and slightly lower than 1 for Discharge; the effect of sex was only weakly significant, and only for Discharge; the HR for Season is greater than 1 for Discharge and lower than 1 for Hospitalization, likely due to fewer severe admissions during the summer.

Our findings also have implications for ED management and health policy. The first concerns the marked differences in the exit process between disease groups. A uniform expansion of hospital capacity may therefore not be the most efficient approach. The pathologies associated with the highest hospitalization cumulative incidence and the longest LoS, namely circulatory and respiratory diseases and blood diseases, point towards the wards where additional inpatient capacity could potentially have the greatest effect on boarding.

A second implication concerns the timing of ED processes. Since the effect of triage was concentrated in the first hours of the stay, monitoring systems based on indices recorded at 24-hour intervals may not fully capture the period in which important decisions are made. The construction of proper indicators based on the estimated survival functions, that distinguish between the discharge and hospitalization pathways, and that account for differences among disease groups, may provide ED managers with more useful information than crude LoS percentiles.

This study has several limitations. The main one is the reduction of the analytic dataset to 10,077 records for computational reasons. Constructing the person-time dataset from all 503,797 eligible accesses would have produced tens of millions of rows and made model estimation impractical, particularly with several non-proportional covariates. We checked that the random sample reproduced the full eligible dataset in terms of the distribution of all model covariates, the two endpoints and the LoS percentiles (S4 and S5 Tables). The estimates, standard errors and Schoenfeld-based proportionality diagnostics were also considered acceptable. Nevertheless, a modelling strategy capable of using the full dataset would improve the standard errors of the estimates and potentially the predictive performance of the model. Developing estimation procedures that can scale to complete administrative datasets therefore remains an important methodological direction for future research. A further limitation concerns frequent users. Because the observation window covered only one year, some repeated attendances by the same patient may not have been captured, which could affect the independence assumptions underlying the patient-level frailty. Finally, the study included only the largest EDs in one region and one calendar year, and considered two of the three possible exits, with Death excluded because of its rarity. The results should therefore be generalised with caution to smaller facilities, other regions or the post-pandemic period.

## Conclusions

This study shows that pathology, age, triage, and season are the covariates associated with ED LoS, with large differences across hospitals and patient groups. Blood and circulatory/respiratory diseases had the longest pre-hospitalization LoS, indicating that overcrowding solutions should be pathology-specific rather than uniform.

The large hospital-level variation, particularly for Hospitalization, highlights the role of institutional factors in driving exit delays. The attenuation of triage effects over time suggests that early interventions for high-acuity patients are critical to reducing prolonged stays.

From a practical perspective, our findings support three targeted actions: (i) developing pathology-specific clinical pathways to speed up admission or discharge decisions for conditions associated with the longest LoS; (ii) implementing early discharge protocols to improve patient flow; and (iii) investigating hospital-specific bottlenecks, particularly for hospitalization processes, to identify whether delays stem from bed shortages, staffing levels, or inefficient clinical pathways. These interventions should be tailored to each hospital’s specific context.

## Supporting information

S1 Fig Estimated ED random effects and 95% CI from the cause-specific frailty models for Discharge. (PDF)

S2 Fig Estimated ED random effects and 95% CI from the cause-specific frailty models for Hospitalization. (PDF)

S3 Fig CIFs and 95% CIs for Discharge. Cardiocirculatory & Respiratory Diseases and Blood Diseases.
(PDF)

S4 Fig CIFs and 95% CIs for Hospitalization. Cardiocirculatory & Respiratory Diseases and Blood Diseases.
(PDF)

S1 Table Number of accesses by Triage and Hospital (row percentages). (DOCX)

S2 Table ICD9 codes aggregation according to homogeneous disease categories. (DOCX)

S3 Table Schoenfeld residual tests, at 95% significance level, for the two separate cause-specific hazard models. (DOCX)

S4 Table Comparison of the sample (n = 10,077) with the full dataset (N = 503,797) by counts and percentages across covariates. (DOCX)

S5 Table Comparison of median LoS (hours) for Discharge and Hospitalization between the sample (n = 10,077) and the full dataset (N = 503,797) across covariates. (DOCX)
