# Supplementary material for: Prolonged length of stay in the Italian emergency departments: A competing risks frailty model
Source: PLoS One. 2026 Nov 11;21(11):e0361888. doi: 10.1371/journal.pone.0361888 (PMC13754151; doi:10.1371/journal.pone.0361888)
Supplement: Supplementary file 1 [file pone.0361888.s001.pdf]

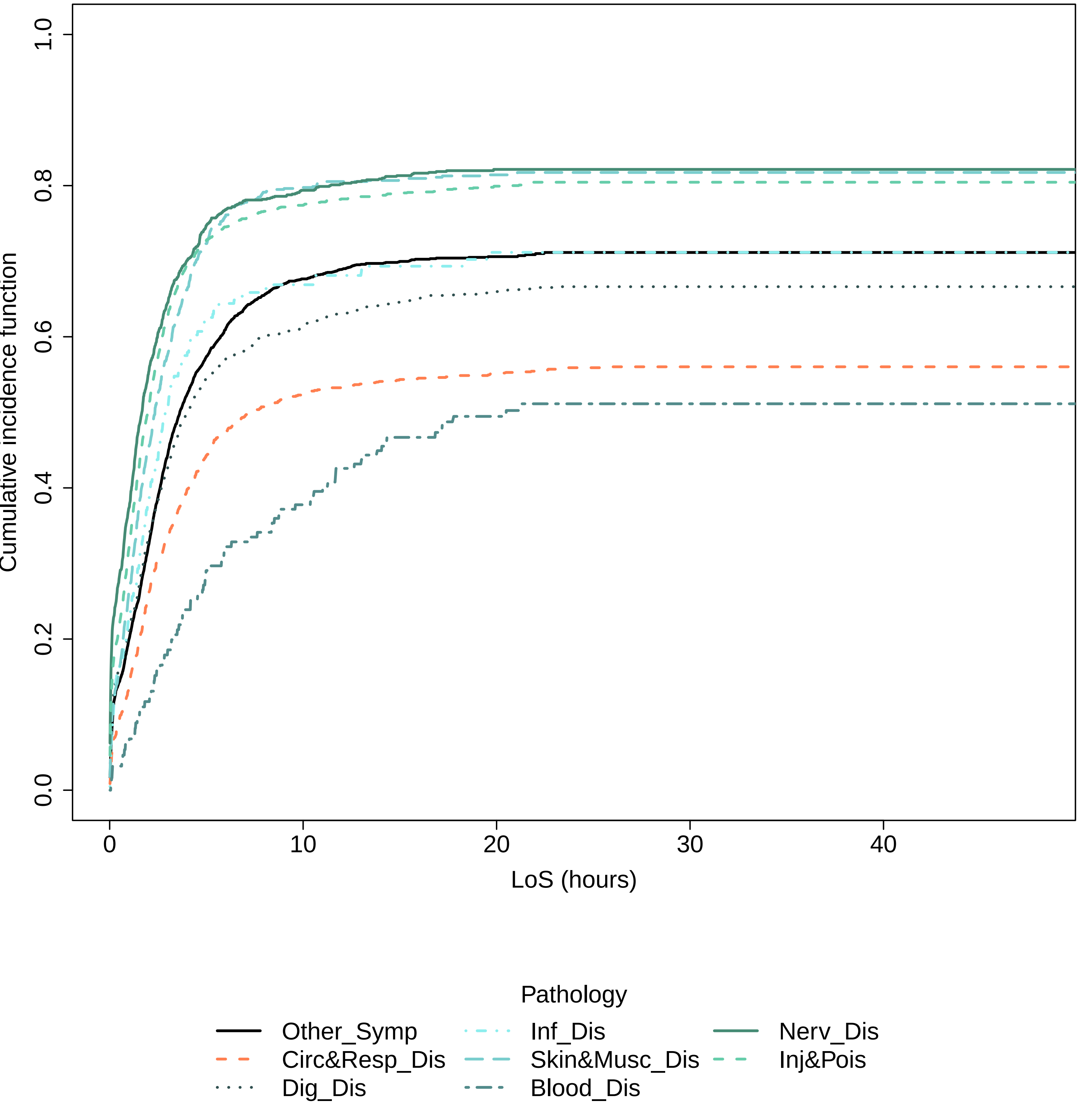

Cumulative incidence function
0.0
0.2
0.4
0.6
0.8
1.0
0
10
20
30
40
LoS (hours)
Pathology
Other_Symp
Circ&Resp_Dis
Dig_Dis
Inf_Dis
Skin&Musc_Dis
Blood_Dis
Nerv_Dis
Inj&Pois
