# Supplementary material for: Prolonged length of stay in the Italian emergency departments: A competing risks frailty model
Source: PLoS One. 2026 Nov 11;21(11):e0361888. doi: 10.1371/journal.pone.0361888 (PMC13754151; doi:10.1371/journal.pone.0361888)
Supplement: Supplementary file 5 [file pone.0361888.s005.docx]

| **Hospital** | **White (%)** | **Green (%)** | **Yellow (%)** | **Red (%)** | **Total (%)** |
| --- | --- | --- | --- | --- | --- |
|  |  |  |  |  |  |
| 1 | 580(3.0) | 10,117(52.0) | 8,327(43.0) | 364(2.0) | 19,388(3.8) |
| 2 | 2,267(9.0) | 14,950(59.0) | 7,396(29.0) | 778(3.0) | 25,391(5.0) |
| 3 | 926(4.5) | 6,694(32.9) | 11,520(56.6) | 1,215(6.0) | 20,355(4.0) |
| 4 | 7,654(24.7) | 9,894(32.0) | 9,939(32.0) | 3,508(11.3) | 30,995(6.2) |
| 5 | 5,431(18.4) | 14,094(47.8) | 9,544(32.4) | 424(1.4) | 29,493(5.9) |
| 6 | 421(1.4) | 17,271(58.8) | 11,402(38.7) | 333(1.1) | 29,427(5.8) |
| 7 | 38(0.1) | 17,976(54.4) | 13,605(41.0) | 1,509(4.6) | 33,128(6.6) |
| 8 | 3,331(9.0) | 16,798(45.5) | 16,045(43.0) | 912(2.5) | 37,086(7.4) |
| 9 | 965(3.0) | 17,593(52) | 14,456(43) | 554(2.0) | 33,568(6.7) |
| 10 | 1,409(4.6) | 22,985(74.3) | 6,350(20.4) | 210(0.7) | 30,954(6.1) |
| 11 | 581(1.5) | 21,381(55.5) | 15,623(40.4) | 1,043(3.0) | 38,628(7.7) |
| 12 | 241(0.9) | 15,304(54.5) | 11,455(40.8) | 1,081(3.8) | 28,081(5.6) |
| 13 | 8,337(17.5) | 22,705(47.5) | 14,416(30.0) | 2,294(5.0) | 47,752(9.5) |
| 14 | 352(1.7) | 10,221(49.6) | 7,674(37.0) | 2,412(11.7) | 20,659(4.1) |
| 15 | 1,299(3.0) | 30,538(72.2) | 9,870(23.4) | 579(1.4) | 42,286(8.4) |
| 16 | 1,376(3.8) | 16,207(44.2) | 17,462(47.7) | 1,561(4.3) | 36,606(7.3) |
| **Total** | **35,208(7.0)** | **264,728(52.0)** | **185,084(37.0)** | **18,777(4.0)** | **503,797(100)** |

**S1 Table**
