# Supplementary material for: Prolonged length of stay in the Italian emergency departments: A competing risks frailty model
Source: PLoS One. 2026 Nov 11;21(11):e0361888. doi: 10.1371/journal.pone.0361888 (PMC13754151; doi:10.1371/journal.pone.0361888)
Supplement: Supplementary file 6 [file pone.0361888.s006.docx]

| **ICD9 Diagnostic codes** | **Description** | **Aggregated ICD9 codes** | **Label** |
| --- | --- | --- | --- |
| 001-139 | Infectious And Parasitic Diseases | 001-139 | Inf_Dis |
| 140-239 | Neoplasms | 140-239 | Neoplasms |
| 240-279 | Endocrine, Nutritional And Metabolic Diseases, And Immunity Disorders | 240-289 | Blood_Dis |
| 280-289 | Diseases Of The Blood And Blood-Forming Organs |  |  |
| 290-319 | Mental Disorders | 290-389 | Nerv_Dis |
| 320-389 | Diseases Of The Nervous System And Sense Organs |  |  |
| 390-459 | Diseases Of The Circulatory System | 390-519 | Circ&Resp_Dis |
| 460-519 | Diseases Of The Respiratory System |  |  |
| 520-579 | Diseases Of The Digestive System | 520-629 | Dig_Dis |
| 580-629 | Diseases Of The Genitourinary System |  |  |
| 630-679 | Complications Of Pregnancy, Childbirth, And The Puerperium | 630-679 | Preg_Comp |
| 680-709 | Diseases Of The Skin And Subcutaneous Tissue | 680-739 | Skin&Musc_Dis |
| 710-739 | Diseases Of The Musculoskeletal System And Connective Tissue |  |  |
| 740-759 | Congenital Anomalies | 740-779 | Cong_Anom |
| 760-779 | Certain Conditions Originating In The Perinatal Period |  |  |
| 780-799 | Symptoms, Signs, And Ill-Defined Conditions | 780-799 + V | Other_Symp |
| V01-V91 | Supplementary Classification Of Factors Influencing Health Status And Contact With Health Services |  |  |
| 800-999 | Injury And Poisoning | 800-999 + E | Inj&Pois |
| E000-E999 | Supplementary Classification Of External Causes Of Injury And Poisoning |  |  |

**S2 Table**
