# Supplementary material for: Prolonged length of stay in the Italian emergency departments: A competing risks frailty model
Source: PLoS One. 2026 Nov 11;21(11):e0361888. doi: 10.1371/journal.pone.0361888 (PMC13754151; doi:10.1371/journal.pone.0361888)
Supplement: Supplementary file 7 [file pone.0361888.s007.docx]

| **Event of interest** | | | | | |
| --- | --- | --- | --- | --- | --- |
| **Discharge** | | | **Hospitalization** | | |
|  | **χ²** | **p-value** |  | **χ²** | **p-value** |
| Age | 1.70 | 0.19 | Age | 5.40 | 0.02 |
| Summer | 6.10 | 0.01 | Summer | 0.66 | 0.88 |
| Red level | 26.10 | <0.001 | Red level | 7.60 | 0.006 |
| Pathology | 126.57 | <0.001 | Pathology | 53 | <0.001 |
| Gender | 4.46 | 0.04 |  |  |  |
| **GLOBAL** | **160.92** | **<0.001** | **GLOBAL** | **57.42** | **<0.001** |

**S3 Table**
