# Supplementary material for: Prolonged length of stay in the Italian emergency departments: A competing risks frailty model
Source: PLoS One. 2026 Nov 11;21(11):e0361888. doi: 10.1371/journal.pone.0361888 (PMC13754151; doi:10.1371/journal.pone.0361888)
Supplement: Supplementary file 8 [file pone.0361888.s008.docx]

| **Comparison Sample vs Population — counts (%) and standardized difference** | | | | | | | | | |
| --- | --- | --- | --- | --- | --- | --- | --- | --- | --- |
|  | **Sample (n=10,077)** | | | **Population (n=503,797)** | | | **Std. diff** | | |
|  | **Discharge** | **Hospitalization** | **Total** | **Discharge** | **Hospitalization** | **Total** | **Discharge** | **Hospitalization** | **Total** |
| ***Gender*** | | | | | | | | | |
| F | 3839(49) | 1169(51) | 5008(49,6) | 192741(49) | 56699(52) | 249440(49,5) | 0 | -0.01 | 0.001 |
| M | 3955(51) | 1114(49) | 5069(50,4) | 201075(51) | 53282(48) | 254357(50,5) | 0 | 0.01 | -0.002 |
| ***Age*** | | | | | | | | | |
| <=30 | 1781(23) | 251(11) | 2023(20) | 90426(23) | 11509(10) | 101935(20) | 0 | 0.05 | 0 |
| 30-50 | 2426(31) | 380(17) | 2806(28) | 122472(31) | 19091(17) | 141563(28) | 0 | 0 | 0 |
| 50-80 | 3001(38) | 1152(50) | 4153(41) | 151765(39) | 54555(50) | 206320(41) | -0.01 | 0 | 0 |
| >80 | 586(7,5) | 500(22) | 1086(11) | 29153(7) | 24826(23) | 53439(11) | -0.07 | 0.022 | 0 |
| ***Pathology*** | | | | | | | | | |
| Infettive | 170(2) | 49(2) | 219(2) | 9176(2) | 2294(2) | 11470(2) | 0 | 0 | 0 |
| Blood | 93(1) | 72(3) | 165(1,6) | 4616(1) | 3404(3) | 8020(1,6) | 0 | 0 | 0. |
| Circ_resp | 608(8) | 555(24) | 1163(12) | 29646(8) | 27243(25) | 56889(11) | 0 | -0.04 | 0.04 |
| Dig_dis | 1071(14) | 409(18) | 1480(15) | 55468(14) | 19557(18) | 75025(16) | 0 | 0 | -0.03 |
| Mental | 1036(13) | 161(7) | 1197(12) | 53623(14) | 7637(7) | 61260(12) | -0.07 | 0 | 0 |
| Skin | 822(11) | 120(5) | 942(9) | 42375(11) | 5226(5) | 47601(9,4) | 0 | 0 | -0.02 |
| Other | 2150(28) | 574(25) | 2724(27) | 109112(28) | 27577(25) | 136689(27) | 0 | 0 | 0 |
| Inj | 1792(23) | 338(16) | 2130(21) | 87715(22) | 16596(15) | 104311(21) | 0.04 | 0.03 | 0 |
| ***Season*** | | | | | | | | | |
| Winter | 1755(23) | 582(25) | 2337(23) | 89364(25) | 27684(25) | 117048(24) | -0.08 | 0 | -0.04 |
| Spring | 1954(25) | 583(26) | 2537(25) | 98273(25) | 27676(25) | 125949(25) | 0 | 0.04 | 0 |
| Summer | 2102(27) | 563(25) | 2665(27) | 106387(27) | 26965(25) | 133352(26) | 0 | 0 | -0.04 |
| Fall | 1982(25) | 555(24) | 2537(25) | 99792(25) | 27656(25) | 127448(25) | 0 | 0.04 | 0 |
| ***Weekday*** | | | | | | | | | |
| Mon-Thu | 4582(59) | 1340(59) | 5922(59) | 234068(59) | 65575(60) | 299643(59) | 0 | 0.02 | 0 |
| Fri-Sun | 3211(41) | 943(41) | 4154(41) | 159748(41) | 44406(40) | 204154(41) | 0 | -0,025 | 0 |
| ***Arrival hour*** | | | | | | | | | |
| 06 am-06 pm | 5140(66) | 1571(69) | 6711(67) | 263913(67) | 76031(69) | 339944(67) | -0.02 | 0 | 0 |
| 06 pm-06 am | 2653(34) | 712(32) | 3365(33) | 129903(33) | 33950(31) | 163853(33) | -0.03 | 0.03 | 0 |
| ***Arrival mode*** | | | | | | | | | |
| Ambulance | 6706(86) | 1358(59) | 8064(80) | 335311(85) | 66773(61) | 402084(80) | 0.01 | 0.03 | 0 |
| Autonomous | 1087(14) | 925(41) | 2012(20) | 58505(15) | 43208(39) | 101713(20) | 0.07 | 0.05 | 0 |
| ***Frequent user*** | | | | | | | | | |
| Yes | 593(8) | 2116(7) | 2709(27) | 31191(8) | 8247(7) | 132925(26) | 0 | 0 | -0.04 |
| No | 7200(92) | 2116(93) | 9316(73) | 361825(92) | 101734(93) | 370072(74) | 0 | 0 | 0.01 |
| ***Triage*** | | | | | | | | | |
| Red | 7747(99) | 1942(88) | 9689(96) | 388152(99) | 96876(88) | 485028(96) | 0 | 0 | 0 |
| Other | 116(1) | 272(12) | 388(4) | 5672(1) | 13105(12) | 18777(4) | 0 | 0 | 0 |
| **Total** | **7794(77)** | **2283(23)** | **10077** | **393816(78)** | **109981(22)** | **503797** | **0.01** | **0.04** |  |
