# Supplementary material for: Prolonged length of stay in the Italian emergency departments: A competing risks frailty model
Source: PLoS One. 2026 Nov 11;21(11):e0361888. doi: 10.1371/journal.pone.0361888 (PMC13754151; doi:10.1371/journal.pone.0361888)
Supplement: Supplementary file 9 [file pone.0361888.s009.docx]

| **Median Length of Stay: Sample vs Population** | | | | | | | |
| --- | --- | --- | --- | --- | --- | --- | --- |
|  | **Sample** | |  | **Population** | | **Mann whitney test p-value** | |
|  | **Discharge** | **Hospitalization** |  | **Discharge** | **Hospitalization** | **Discharge** | **Hospitalization** |
| ***Gender*** | | | | | | | |
| F | 1.72 | 3.45 |  | 1.68 | 3.87 | 0.45 | 0.71 |
| M | 1.82 | 3.54 |  | 1.73 | 3.68 | 0.83 | 0.70 |
| ***Age*** | | | | | | | |
| <=30 | 1.40 | 2.37 |  | 1.33 | 2.43 | 0.46 | 0.62 |
| 30-50 | 1.45 | 2.72 |  | 1.43 | 2.70 | 0.51 | 0.54 |
| 50-80 | 2.08 | 3.73 |  | 2.02 | 4.15 | 0.92 | 0.77 |
| >80 | 3.25 | 5.07 |  | 3.12 | 5.22 | 0.17 | 0.28 |
| ***Pathology*** | | | | | | | |
| Inf_Dis | 1.84 | 3.27 |  | 1.73 | 4.00 | 0.53 | 0.75 |
| Blood_Dis | 7.18 | 7.56 |  | 6.48 | 6.53 | 0.97 | 0.31 |
| Circ_resp | 2.63 | 5.38 |  | 2.60 | 5.13 | 0.60 | 0.85 |
| Dig_dis | 2.20 | 4.40 |  | 2.02 | 4.10 | 0.09 | 0.97 |
| Skin_Dis | 1.57 | 3.88 |  | 1.55 | 3.83 | 0.78 | 0.28 |
| Other_symp | 2.37 | 3.42 |  | 2.13 | 3.27 | 0.25 | 0.11 |
| Inj_pois | 1.33 | 3.10 |  | 1.33 | 3.20 | 0.42 | 0.88 |
| ***Season*** | | | | | | | |
| Winter | 1.85 | 3.95 |  | 1.80 | 4.10 | 0.54 | 0.10 |
| Spring | 1.68 | 3.60 |  | 1.70 | 3.75 | 0.18 | 0.52 |
| Summer | 1.65 | 3.18 |  | 1.62 | 3.50 | 0.20 | 0.10 |
| Fall | 1.82 | 3.45 |  | 1.72 | 3.77 | 0.66 | 0.99 |
| ***Weekday*** | | | | | | | |
| Mon-Thu | 1.78 | 3.55 |  | 1.73 | 3.85 | 0.14 | 0.76 |
| Fri-Sun | 1.73 | 3.45 |  | 1.67 | 3.65 | 0.22 | 0.73 |
| ***Arrival hour*** | | | | | | | |
| 06 am-06 pm | 1.98 | 4.14 |  | 1.85 | 4.23 | 0.87 | 0.58 |
| 06 pm-06 am | 1.68 | 3.20 |  | 1.65 | 3.55 | 0.72 | 0.66 |
| ***Arrival mode*** | | | | | | | |
| Ambulance | 3.22 | 4.47 |  | 3.07 | 4.87 | 0.37 | 0.50 |
| Autonomous | 1.62 | 3.12 |  | 1.53 | 3.25 | 0.37 | 0.56 |
| ***Frequent user*** | | | | | | | |
| Yes | 1.67 | 3.27 |  | 1.68 | 3.77 | 0.97 | 0.78 |
| No | 1.77 | 3.53 |  | 1.70 | 3.77 | 0.21 | 0.29 |
| ***Triage*** | | | | | | | |
| Red | 6.15 | 3.77 |  | 6.90 | 3.80 | 0.75 | 0.99 |
| Other | 1.68 | 3.88 |  | 1.70 | 3.87 | 0.50 | 0.99 |
| **Total** | **1.77** | **3.50** |  | **1.70** | **3.77** | **0.71** | **0.97** |
